# Supplementary material for: An unsupervised deep learning framework for predicting human essential genes from population and functional genomic data
Source: BMC Bioinformatics. 2023 Sep 18;24:347. doi: 10.1186/s12859-023-05481-z (PMC10506225; doi:10.1186/s12859-023-05481-z)
Supplement: Supplementary file 1 — Additional file 1. Supplemental file containing: Supplementary Fig. 1 depicting the performance of DeepLOF and alternative methods in predicting dominant negative genes, Supplementary Table 1 which provides a detailed description of the genomic features used for model training, and Supplementary Table 2 which shows the statistical significance of the differences in AUC between DeepLOF and alternative methods in predicting essential genes. [file 12859_2023_5481_MOESM1_ESM.pdf]

Supplementary material for:

An unsupervised deep learning framework for predicting human essential genes from population and functional genomic data

Troy M. LaPolice[1,2,3*], Yi-Fei Huang[1,3*]

[1]Department of Biology, Pennsylvania State University, University Park, PA 16802, USA

[2]Bioinformatics and Genomics Graduate Program, Pennsylvania State University, University Park, PA 16802, USA

[3]Huck Institutes of the Life Sciences, Pennsylvania State University, University Park, PA 16802, USA

*Correspondence to: troy.lapolice@psu.edu, yuh371@psu.edu

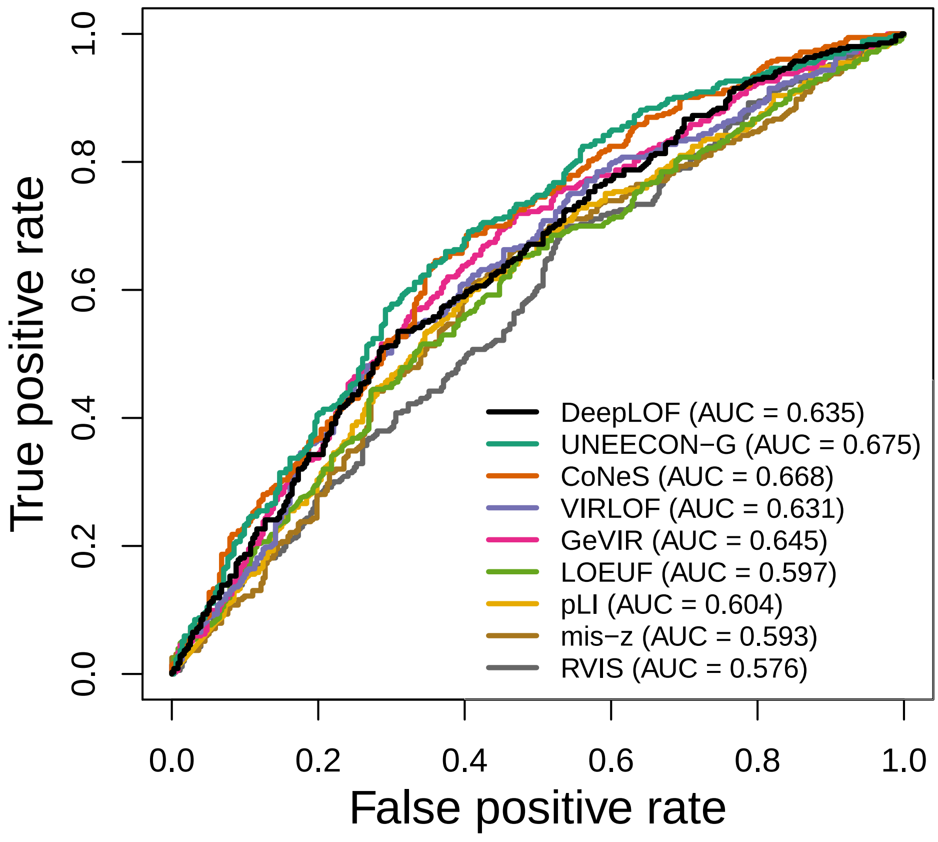

Supplementary Fig. 1: Performance of DeepLOF and alternative methods in predicting dominant negative genes.

Supplementary Table 1: Genomic features for model training.

Feature name	Feature type	Log transformation	Data source
H3K9ac signal	Continuous	Yes	[1]
H3K27me3 signal	Continuous	Yes	[1]
H3K4me3 signal	Continuous	Yes	[1]
H2A.Z signal	Continuous	Yes	[1]
Enhancer number	Continuous	Yes	[1]
GO embryo development	Binary	N/A	[2]
GO central nervous development	Binary	N/A	[2]
Reactome nervous system development	Binary	N/A	[2]
Reactome developmental biology	Binary	N/A	[2]
Transcription factor	Binary	N/A	[3]
Protein complex	Binary	N/A	[4]
Promoter CpG density	Continuous	No	[5]
Promoter phastCons score	Continuous	No	[5]
Exonic phastCons score	Continuous	No	[5]
Mean expression level	Continuous	Yes	[6]
Tissue specificity (tau)	Continuous	No	[6]
PPI degree	Continuous	Yes	[6]
UNEECON-G	Continuous	No	[7]

Supplementary Table 2: Statistical significance of the differences in AUC between DeepLOF and alternative methods in predicting essential genes. The numbers represent P-values from the DeLong test. *: $P < 0.05$; **: $P < 0.01$; ***: $P < 0.001$.

DeepLOF	ClinGen haploinsufficient genes	Human orthologs of mouse essential genes	Human essential genes in cell lines
vs. UNEECON-G	1.332e-05 ***	5.835e-04 ***	5.577e-10 ***
vs. CoNeS	8.679e-04 ***	3.688e-03 **	4.068e-21 ***
vs. VIRLOF	6.909e-07 ***	4.766e-06 ***	1.260e-02 *
vs. GeVIR	1.965e-08 ***	2.181e-05 ***	7.913e-05 ***
vs. LOEUF	6.601e-10 ***	1.073e-09 ***	3.151e-12 ***
vs. pLI	1.090e-09 ***	3.263e-10 ***	6.640e-50 ***
vs. mis-z	8.264e-22 ***	1.435e-14 ***	5.962e-09 ***
vs. RVIS	8.893e-23 ***	1.834e-16 ***	2.067e-15 ***

References

[1] Han, X., Chen, S., Flynn, E., Wu, S., Wintner, D., Shen, Y.: Distinct epigenomic patterns are associated with haploinsufficiency and predict risk genes of developmental disorders. Nature Communications **9**(1), 2138 (2018)

[2] Liberzon, A., Subramanian, A., Pinchback, R., Thorvaldsdóttir, H., Tamayo, P., Mesirov, J.P.: Molecular signatures database (MSigDB) 3.0. Bioinformatics **27**(12), 1739–1740 (2011)

[3] Barrera, L.A., Vedenko, A., Kurland, J.V., Rogers, J.M., Gisselbrecht, S.S., Rossin, E.J., Woodard, J., Mariani, L., Kock, K.H., Inukai, S., Siggers, T., Shokri, L., Gordân, R., Sahni, N., Cotsapas, C., Hao, T., Yi, S., Kellis, M., Daly, M.J., Vidal, M., Hill, D.E., Bulyk, M.L.: Survey of variation in human transcription factors reveals prevalent dna binding changes. Science **351**(6280), 1450–1454 (2016)

[4] Giurgiu, M., Reinhard, J., Brauner, B., Dunger-Kaltenbach, I., Fobo, G., Frishman, G., Montrone, C., Ruepp, A.: Corum: the comprehensive resource of mammalian protein complexes–2019. Nucleic Acids Res **47**(D1), 559–563 (2019)

[5] Boukas, L., Bjornsson, H.T., Hansen, K.D.: Promoter CpG density predicts downstream gene loss-of-function intolerance. American Journal of Human Genetics **107**(3), 487–498 (2020)

[6] Huang, Y.-F.: Dissecting genomic determinants of positive selection with an evolution-guided regression model. Molecular Biology and Evolution **39**(1), 291 (2022)

[7] Huang, Y.-F.: Unified inference of missense variant effects and gene constraints in the human genome. PLOS Genetics **16**(7), 1008922 (2020)
